# Supplementary material for: The chitin synthase regulator CSR-3 promotes cellular integrity during cell-cell fusion in the filamentous ascomycete fungus Neurospora crassa
Source: PLoS Genet. 2025 Oct 10;21(10):e1011891. doi: 10.1371/journal.pgen.1011891 (PMC12561907; doi:10.1371/journal.pgen.1011891)
Supplement: S1 Literature — (PDF) [file pgen.1011891.s001.pdf]

Literature for Supplementary Materials

Colot, H. V., G. Park, G. E. Turner, C. Ringelberg, C. M. Crew *et al.*, 2006 A high-throughput gene knockout procedure for Neurospora reveals functions for multiple transcription factors. Proc Natl Acad Sci U S A 103**:** 10352-10357.

Dettmann, A., Y. Heilig, S. Ludwig, K. Schmitt, J. Illgen *et al.*, 2013 HAM-2 and HAM-3 are central for the assembly of the Neurospora STRIPAK complex at the nuclear envelope and regulate nuclear accumulation of the MAP kinase MAK-1 in a MAK-2-dependent manner. Mol Microbiol 90**:** 796-812.

Fajardo-Somera, R. A., B. Johnk, O. Bayram, O. Valerius, G. H. Braus *et al.*, 2015 Dissecting the function of the different chitin synthases in vegetative growth and sexual development in Neurospora crassa. Fungal Genet Biol 75**:** 30-45.

Fleissner, A., and N. L. Glass, 2007 SO, a protein involved in hyphal fusion in *Neurospora crassa*, localizes to septal plugs. Eukaryot Cell 6**:** 84-94.

Fleissner, A., A. C. Leeder, M. G. Roca, N. D. Read and N. L. Glass, 2009 Oscillatory recruitment of signaling proteins to cell tips promotes coordinated behavior during cell fusion. Proc Natl Acad Sci U S A 106**:** 19387-19392.

Freitag, M., P. C. Hickey, N. B. Raju, E. U. Selker and N. D. Read, 2004 GFP as a tool to analyze the organization, dynamics and function of nuclei and microtubules in Neurospora crassa. Fungal Genet Biol 41**:** 897-910.

Jonkers, W., A. C. Leeder, C. Ansong, Y. Wang, F. Yang *et al.*, 2014 HAM-5 functions as a MAP kinase scaffold during cell fusion in Neurospora crassa. PLoS Genet 10**:** e1004783.

Lu, S., J. Wang, F. Chitsaz, M. K. Derbyshire, R. C. Geer *et al.*, 2020 CDD/SPARCLE: the conserved domain database in 2020. Nucleic Acids Res 48**:** D265-D268.

Maurer-Stroh, S., and F. Eisenhaber, 2005 Refinement and prediction of protein prenylation motifs. Genome Biol 6**:** R55.

Riquelme, M., S. Bartnicki-Garcia, J. M. Gonzalez-Prieto, E. Sanchez-Leon, J. A. Verdin-Ramos *et al.*, 2007 Spitzenkorper localization and intracellular traffic of green fluorescent protein-labeled CHS-3 and CHS-6 chitin synthases in living hyphae of *Neurospora crassa*. Eukaryot Cell 6**:** 1853-1864.

Schürg, T., U. Brandt, C. Adis and A. Fleissner, 2012 The Saccharomyces cerevisiae BEM1 homologue in Neurospora crassa promotes co-ordinated cell behaviour resulting in cell fusion. Mol Microbiol 86**:** 349-366.

Verdin, J., S. Bartnicki-Garcia and M. Riquelme, 2009 Functional stratification of the Spitzenkorper of Neurospora crassa. Mol Microbiol 74**:** 1044-1053.

Weichert, M., A. Lichius, B. E. Priegnitz, U. Brandt, J. Gottschalk *et al.*, 2016 Accumulation of specific sterol precursors targets a MAP kinase signaling pathway mediating cell-cell recognition and fusion. PNAS 113**:** 11877-11882.
