# Supplementary material for: The chitin synthase regulator CSR-3 promotes cellular integrity during cell-cell fusion in the filamentous ascomycete fungus Neurospora crassa
Source: PLoS Genet. 2025 Oct 10;21(10):e1011891. doi: 10.1371/journal.pgen.1011891 (PMC12561907; doi:10.1371/journal.pgen.1011891)
Supplement: S1 Fig — (A) Subcellular localization of GFP-CSA-1 in fusing germlings over time (strain SH_250: Pccg-1-gfp-csa-1). (B) Subcellular localization of GFP-CSA-2 in fusing germlings of strain SH_248 (Pccg-1-gfp-csa-2) over time. (C) Localization of GFP-CSR-3 (arrow head) in strain SH_45 (Pccg-1-gfp-csr-3) during germling fusion. Scale bars = 10 µm. Time scale = minutes. (PDF) [file pgen.1011891.s002.pdf]

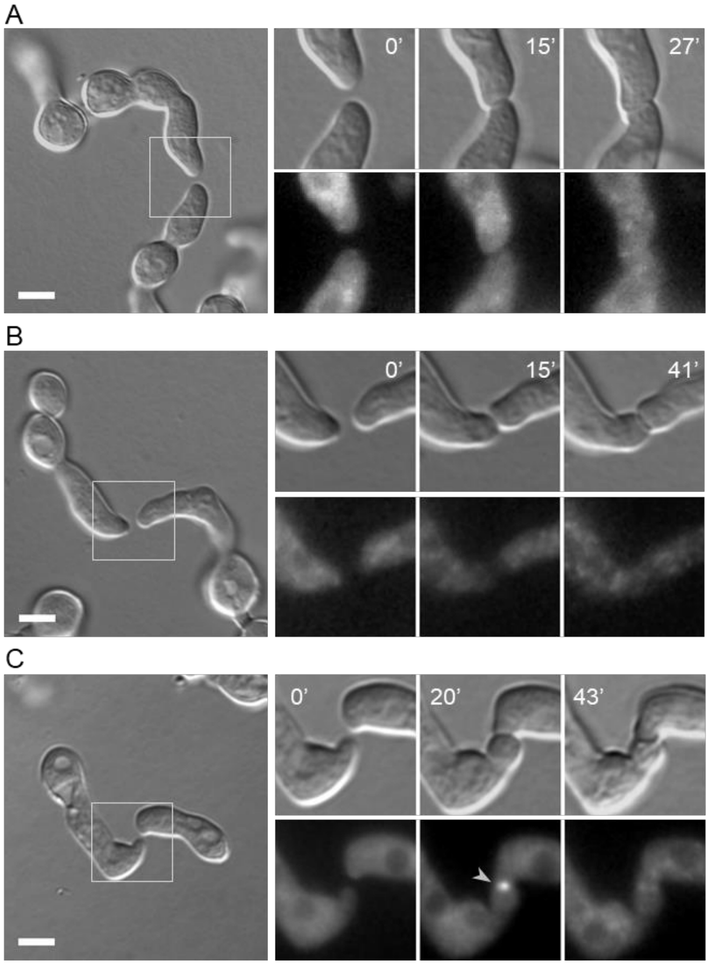

S1 Fig: The chitin synthase regulator 3 accumulates at the contact sites of fusing *N. crassa* germlings.

(A) Subcellular localization of GFP-CSA-1 in fusing germlings over time (strain SH_250: *Pccg-1-gfp-csa-1*). (**B)** Subcellular localization of GFP-CSA-2 in fusing germlings of strain SH_248 (*Pccg-1-gfp-csa-2*) over time. **(C)** Localization of GFP-CSR-3 (arrow head) in strain SH_45 (*Pccg-1-gfp-csr-3*) during germling fusion. Scale bars = 10 µm. Time scale = minutes.
