# Supplementary material for: The chitin synthase regulator CSR-3 promotes cellular integrity during cell-cell fusion in the filamentous ascomycete fungus Neurospora crassa
Source: PLoS Genet. 2025 Oct 10;21(10):e1011891. doi: 10.1371/journal.pgen.1011891 (PMC12561907; doi:10.1371/journal.pgen.1011891)
Supplement: S2 Fig — (A) Scheme of the CSR-3 protein with selected motifs. Domains were predicted using CDSEARCH/cdd-analysis from NCBI (conserved domain database CDD; NCBI;) (Lu et al. 2020) (grey). The three selected phosphorylation sites (green) are from a previous phosphoproteome study done with iTRAQ (Jonkers et al. 2014) and the prediction for prenylation motifs (blue) were performed by using PrePS (Prenylation Prediction Suite) (Maurer-Stroh and Eisenhaber 2005). (B/C) Southern blot analysis of csr-3 deletion in strain FGSC 9719: genomic DNA from eight primary transformants and the recipient strain FGSC 9719 was digested with EcoRI and subjected to southern blotting. Hybridization was performed using the radioactively labeled entire gene replacement cassette. Detection of signals at around 5 and 7 kb indicate a successful replacement of csr-3 with the hph knock-out cassette. A signal at 15 kb indicates non-transformed nuclei. The 4100 bp signal in the recipient strain and transformants results from hybridization of the trpC promoter in the probe - driving expression of the hygromycin resistance gene - with the phosphinothricin resistance cassette, which contains the same promoter and is integrated at the mus52 locus. The asterisk marks the primary transformant used for purification by crossing, resulting in homokaryotic csr-3 deletion mutants GN5-20 and GN5-21. (PDF) [file pgen.1011891.s003.pdf]

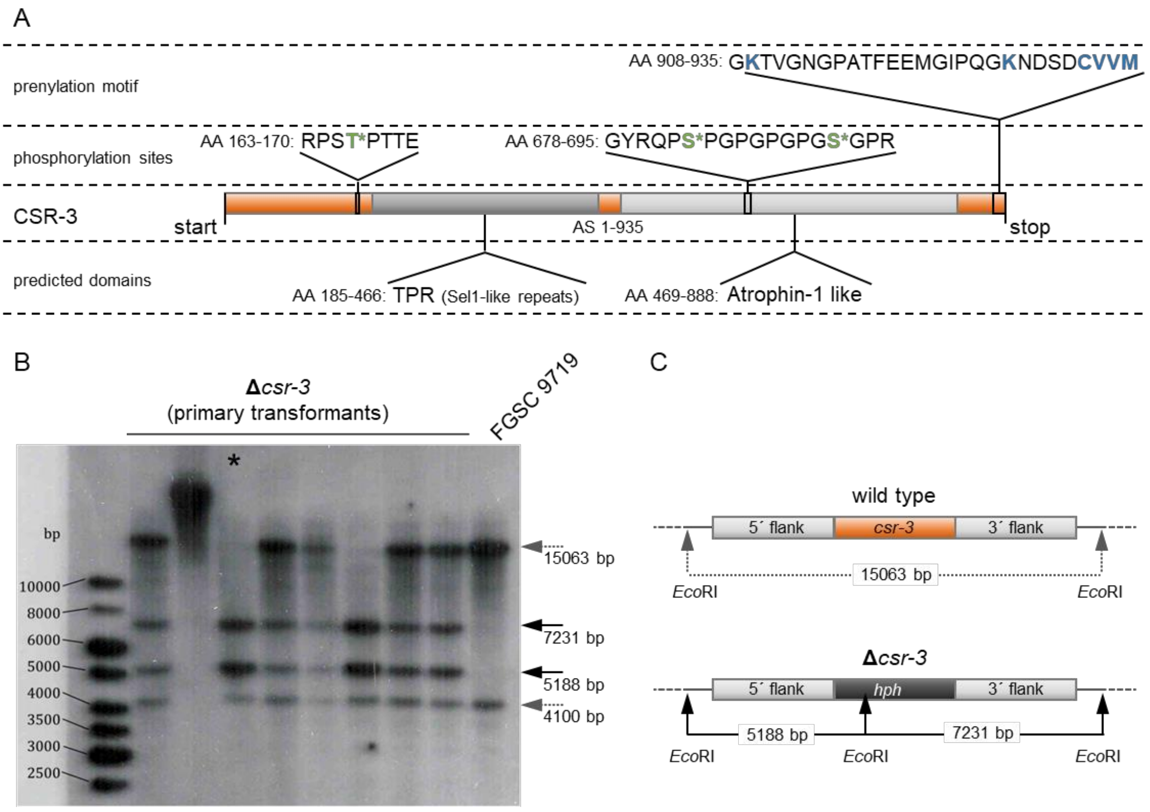

S2 Fig: CSR-3 exhibits multiple conserved motifs and validation of the created *csr-3* deletion mutant by southern blot.

(A) Scheme of the CSR-3 protein with selected motifs. Domains were predicted using CDSAERCH/cdd-analysis from NCBI (conserved domain database CDD; NCBI;) (LU *et al.* 2020) (grey). The three selected phosphorylation sites (green) are from a previous phosphoproteom study done with iTRAQ (JONKERS *et al.* 2014) and the prediction for prenylation motifs (blue) were performed by using PrePS (Prenylation Prediction Suite) (MAURER-STROH AND EISENHABER 2005). **(B)** Southern blot analysis of *csr-3* deletion in strain FGSC 9719: genomic DNA from eight primary transformants and the recipient strain FGSC 9719 was digested with *Eco*RI and subjected to southern blotting. Detection of signals at around 5 and 7 kb indicate a successful replacement of *csr-3* with the *hph* knock-out cassette. A signal at 15 kb indicates non-transformed nuclei. Due to the *mus52* deletion in strain FGSC 9719, all transformants exhibit a signal at 4100 bp. The asterisk marks the primary transformant used for purification by crossing, resulting in homokaryotic *csr-3* deletion mutants GN5-20 and GN5-21.
