# Supplementary material for: The chitin synthase regulator CSR-3 promotes cellular integrity during cell-cell fusion in the filamentous ascomycete fungus Neurospora crassa
Source: PLoS Genet. 2025 Oct 10;21(10):e1011891. doi: 10.1371/journal.pgen.1011891 (PMC12561907; doi:10.1371/journal.pgen.1011891)
Supplement: S3 Fig — (A) Mature colonies of wild type (N1-02) and the Δcsr-3 mutant (GN5-21) on agar plates (left) and details of the hyphal front (FGSC 2489; GN5-20) (right). (B, C) Macroscopic phenotype of wild type (FGSC 2489) and Δcsr-3 mutants (GN5-20, SH_283, SH_290, SH_125). Complemented strains (SH_283: Pcsr-3-gfp-csr-3, Δcsr-3; SH_125: Pccg-1-gfp-csr-3, Δcsr-3) show no phenotypic differences from the reference strain whereas mutants (GN5-20: Δcsr-3; SH_290: Pcsr-3-gfp-csr-3SAAX, Δcsr-3) lacking or with non-functional CSR-3 exhibit a brownish pigmentation (arrows in enlarged selection) and reduced linear growth linear growth. (D, E) Csr-3 deletion has no effect on conidiation or aerial hyphae length. Statistically significant differences (p ≤ 0.05) are indicated by asterisks. For details of quantification, see materials and methods. (PDF) [file pgen.1011891.s004.pdf]

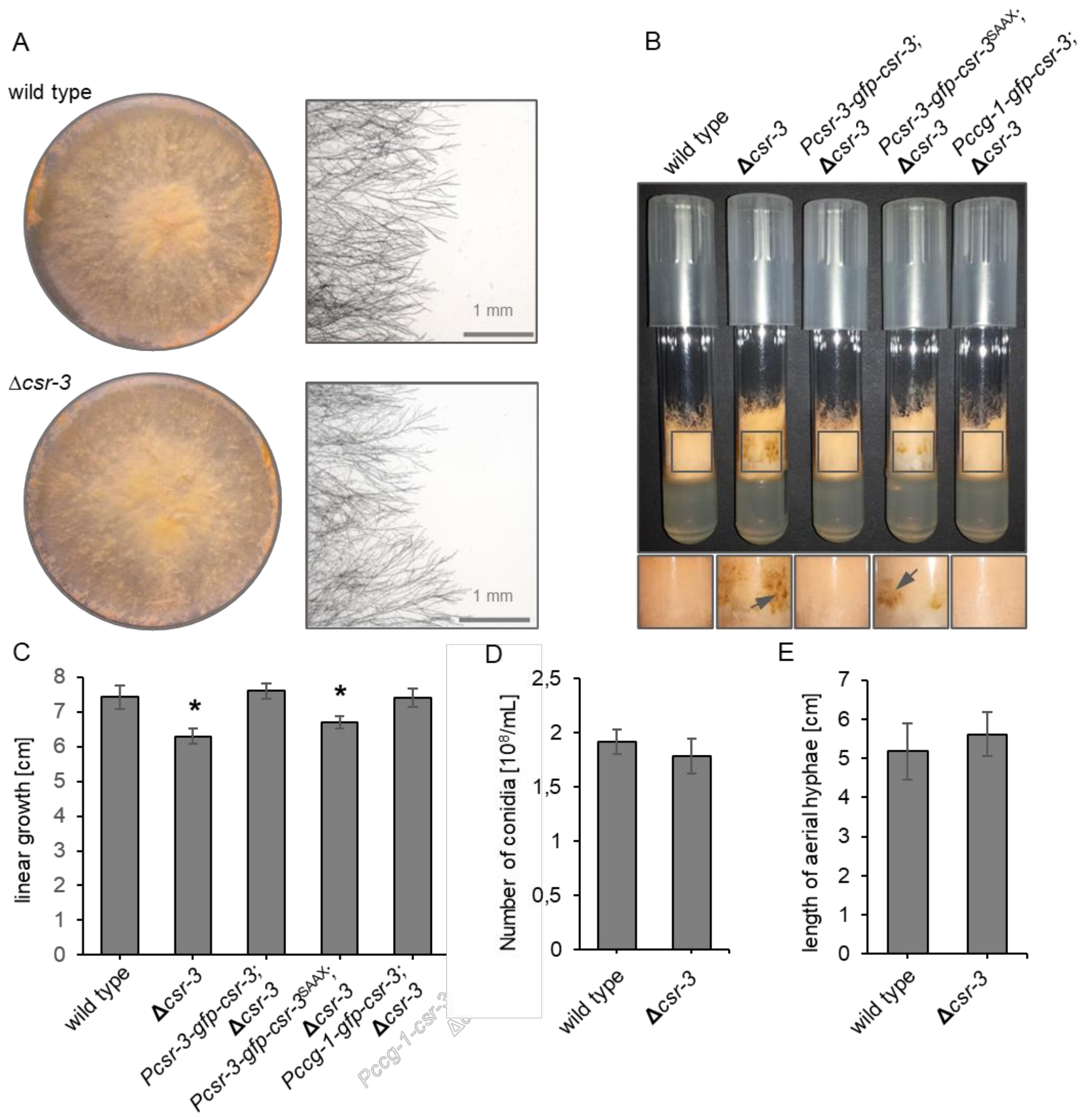


**S3 Fig: Impact of CSR-3 on vegetative colony establishment.**

**(A)** Mature colonies of wild type (N1-02) and the Δ*csr-3* mutant (GN5-21) on agar plates (left) and details of the hyphal front (FGSC 2489; GN5-20) (right). **(B, C)** Macroscopic phenotype of wild type (FGSC 2489) and Δ*csr-3* mutants (GN5-20, SH_283, SH_290, SH_125). Complemented strains (SH_283: *Pcsr-3-gfp-csr-3,* Δ*csr-3,* SH_125: *Pccg-1-gfp-csr-3,* Δ*csr-3*) show no phenotypic differences from the reference strain whereas mutants (GN5-20: Δ*csr-3*; SH_290: *Pcsr-3-gfp-csr-3*$^{SAAX}$, Δ*csr-3*) lacking or with non-functional CSR-3 exhibit a brownish pigmentation (arrows in enlarged selection) and reduced linear growth linear growth. **(D, E)** *Csr-3* deletion has no effect on conidiation or aerial hyphae length. Statistically significant differences ($p \leq 0.05$) are indicated by asterisks. For details of quantification, see materials and methods.
