# Supplementary material for: The chitin synthase regulator CSR-3 promotes cellular integrity during cell-cell fusion in the filamentous ascomycete fungus Neurospora crassa
Source: PLoS Genet. 2025 Oct 10;21(10):e1011891. doi: 10.1371/journal.pgen.1011891 (PMC12561907; doi:10.1371/journal.pgen.1011891)
Supplement: S4 Fig — (A) Time-lapse imaging at 1–4 h temporal resolution of germling populations of the wild-type strain (FGSC 2489) and the csr-3 deletion mutant (GN5-20) on MM medium at 30°C. Scale bars = 20 µm. (B) Quantification of germination and tropic interaction at four different time points during incubation of the wild type strain (FGSC 2489) and the csr-3 deletion mutant (GN5-20) on MM medium at 30°C revealed no statistically significant differences. n = 100 spores each. (PDF) [file pgen.1011891.s005.pdf]

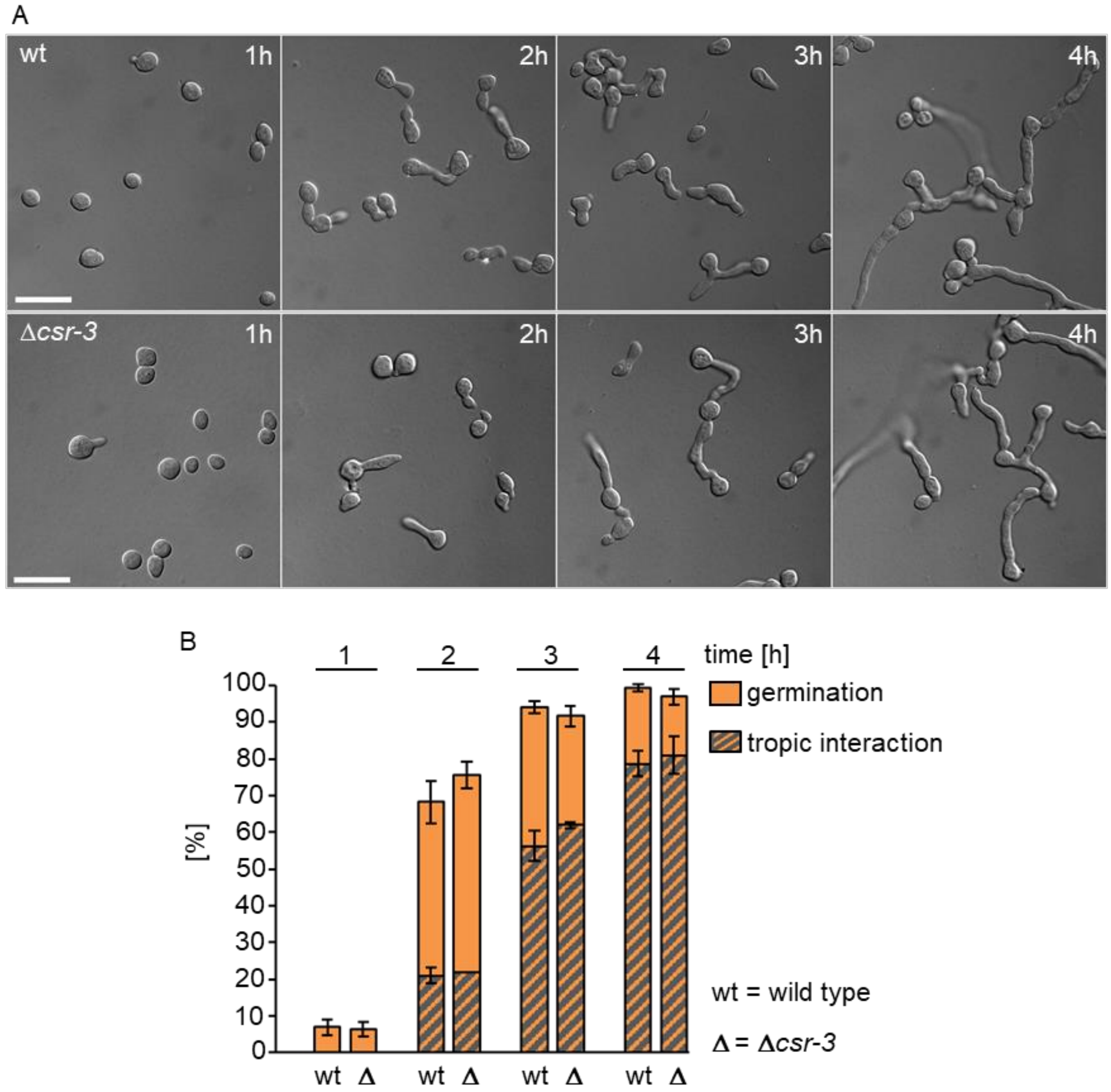

S4 Fig: CSR-3 is dispensable for germination, tropic interaction and fusion rate in germlings.

(A) Time-lapse imaging at 1-4 h temporal resolution of germling populations of the wild-type strain (FGSC 2489) and the *csr-3* deletion mutant (GN5-20) on MM medium at 30°C. Scale bars = 20 µm. **(B)** Quantification of germination and tropic interaction at four different time points during incubation of the wild type strain (FGSC 2489) and the *csr-3* deletion mutant (GN5-20) on MM medium at 30°C revealed no statistically significant differences. n = 100 spores each
