# Supplementary material for: The chitin synthase regulator CSR-3 promotes cellular integrity during cell-cell fusion in the filamentous ascomycete fungus Neurospora crassa
Source: PLoS Genet. 2025 Oct 10;21(10):e1011891. doi: 10.1371/journal.pgen.1011891 (PMC12561907; doi:10.1371/journal.pgen.1011891)
Supplement: S5 Fig — (A) Subcellular localization of GFP-CSR-3 (arrow heads) during germling fusion in strain SH_285 (Pcsr-3-1-gfp-csr-3, Δcsr-3). (B) Localization GFP-CSR-3 during septum formation in a hypha of strain SH_285 (Pcsr-3-1-gfp-csr-3, Δcsr-3). Scale bar = 5 µm (A), 2 µm (B). Time scale = minutes. (PDF) [file pgen.1011891.s006.pdf]

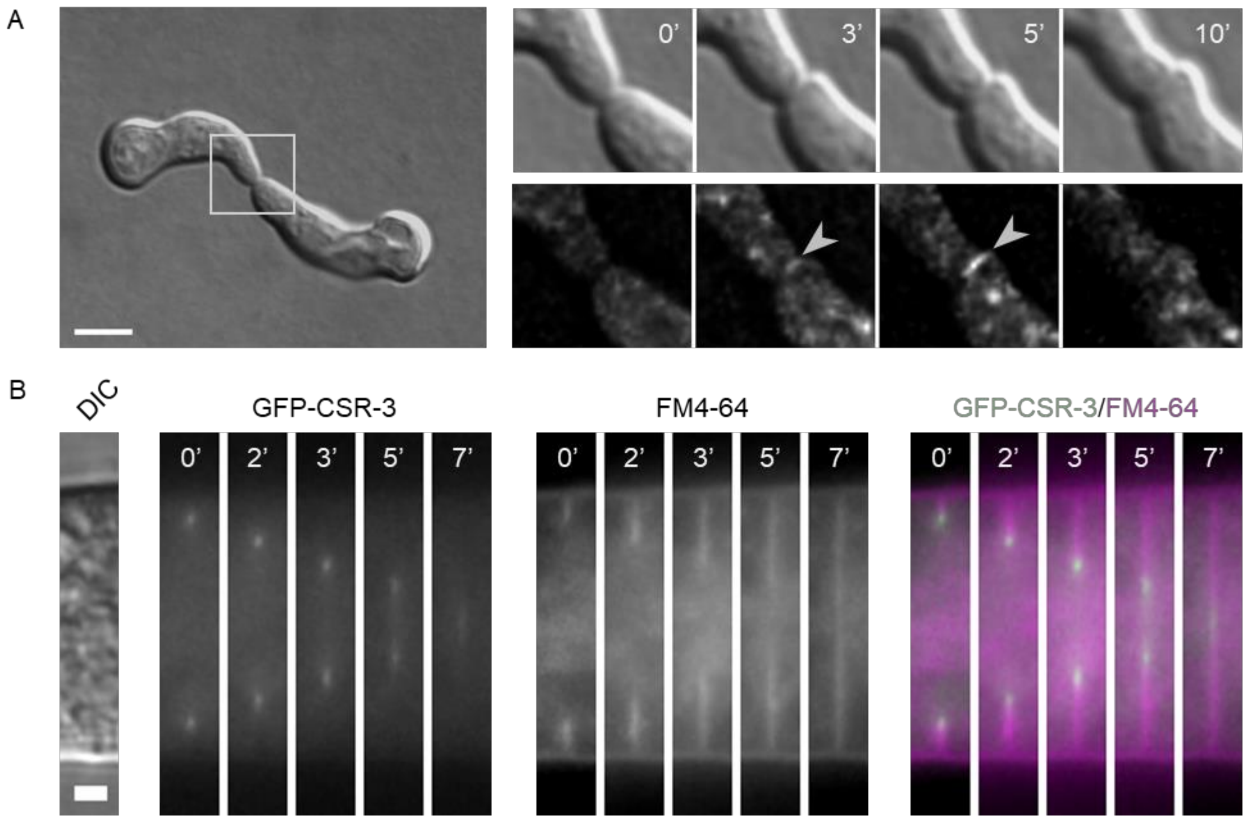

S5 Fig: Recruitment of GFP-CSR-3 under control of the native promotor in germlings and hyphae.

(A) Subcellular localization of GFP-CSR-3 (arrow heads) during germling fusion in strain SH_285 (*Pcsr-3-1-gfp-csr-3*, Δ*csr-3*). **(B)** Localization GFP-CSR-3 during septum formation in a hypha of strain SH_285 (*Pcsr-3-1-gfp-csr-3*, Δ*csr-3*). Scale bar = 5 μm (A), 2 μm (B). Time scale = minutes.
