# Supplementary material for: The chitin synthase regulator CSR-3 promotes cellular integrity during cell-cell fusion in the filamentous ascomycete fungus Neurospora crassa
Source: PLoS Genet. 2025 Oct 10;21(10):e1011891. doi: 10.1371/journal.pgen.1011891 (PMC12561907; doi:10.1371/journal.pgen.1011891)
Supplement: S6 Fig — (A) Sample illustration of examined sections and septa of vegetative hyphae (here wild type strain FGSC 2489). (B) Strains were stained with CFW to visualize the cell wall to measure the distance between and the diameter of septa. (C) Quantification of time needed to form a complete septum in the wild type strain (FGSC 2489) and the strain GN5-20 (Δcsr-3). (D) Quantification of germ tube length and septa at three time points in wild type (FGSC 2489) and csr-3 deletion mutant (GN5-20) germling populations. Error bars represent the standard deviation calculated from at least three independent experiments (B,C: n = 6–15 hyphae each; D: n = 55–140 germlings each). Statistically significant differences (p ≤ 0.05) are indicated by asterisks. (PDF) [file pgen.1011891.s007.pdf]

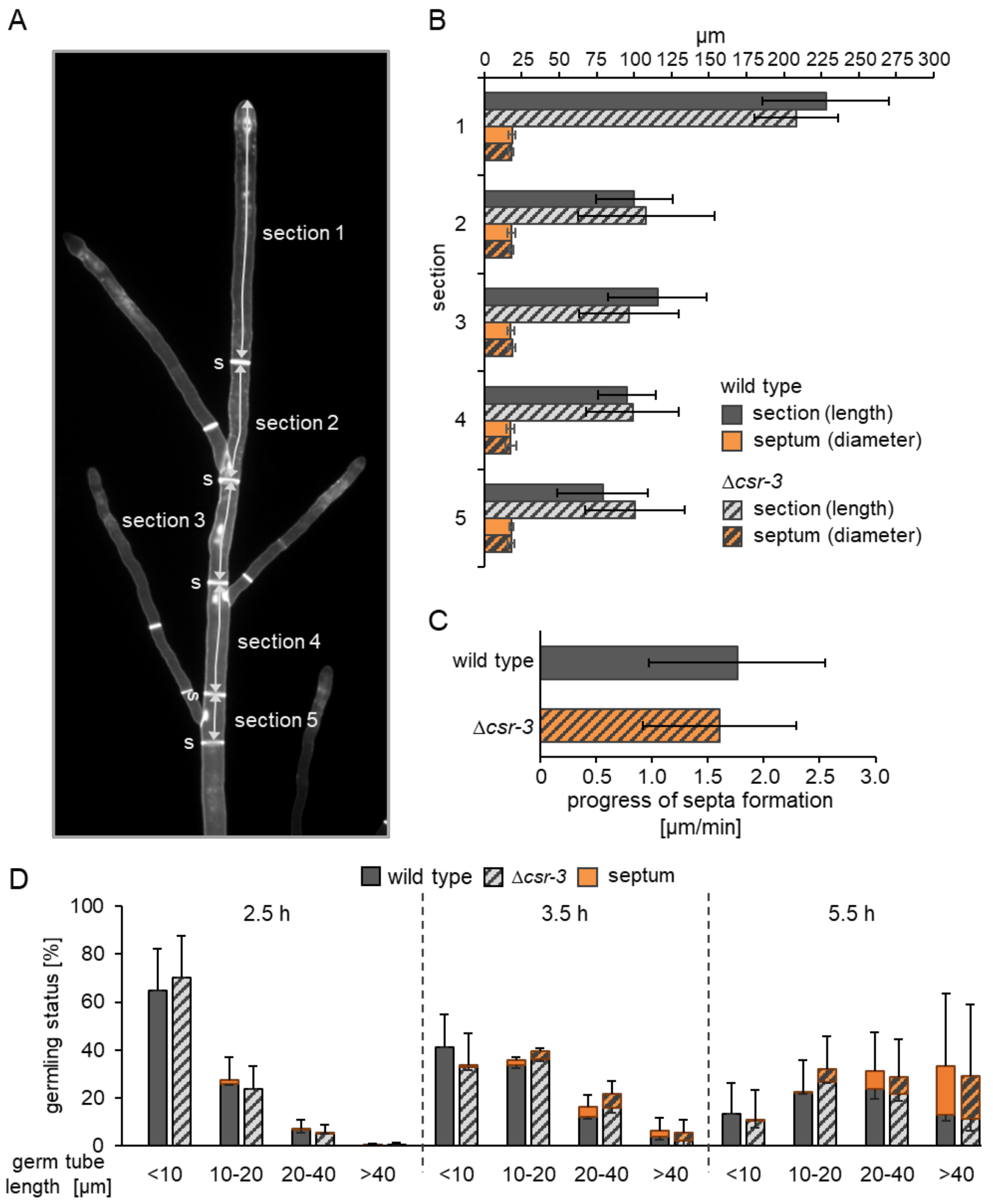

S6 Fig: CSR-3 is dispensable for the development of vegetative hyphae.

(A) Sample illustration of examined sections and septa of vegetative hyphae (here wild type strain FGSC 2489). **(B)** Strains were stained with CFW to visualize the cell wall to measure the distance between and the diameter of septa. **(C)** Quantification of time needed to form a complete septum in the wild type strain (FGSC 2489) and the strain GN5-20 (Δ*csr-3*). **(D)** Quantification of germ tube length and septa at three time points in wild type (FGSC 2489) and *csr-3* deletion mutant (GN5-20) germling populations. Error bars represent the standard deviation calculated from at least three independent experiments (B,C: n = 6-15 hyphae each; D: n= 55-140 germlings each). Statistically significant differences ($p \leq 0.05$) are indicated by asterisks.
