# Supplementary material for: The chitin synthase regulator CSR-3 promotes cellular integrity during cell-cell fusion in the filamentous ascomycete fungus Neurospora crassa
Source: PLoS Genet. 2025 Oct 10;21(10):e1011891. doi: 10.1371/journal.pgen.1011891 (PMC12561907; doi:10.1371/journal.pgen.1011891)
Supplement: S7 Fig — (A/B) Subcellular localization of GFP-CSR-3 (SH_125: Pccg-1-gfp-csr-3, Δcsr-3) (arrow heads) during the formation of macroconidia within an early (A) and a late (B) time point. (C) Illustration of the localization pattern of CSR-3 during the development of asexual macroconidia. (D) Localization of GFP-CSR-3 (arrow heads) during microconidiation. (E) CSR-3 is recruited to the conidiation site (0’) and released (3’) during microconidium formation (F) Illustration of the localization pattern of C SR-3 during the development of asexual microconidia. Strains were grown on plates with water agar. Scale bars = 10 µm (overview) and 2 µm (insets). Time scale = minutes. (PDF) [file pgen.1011891.s008.pdf]

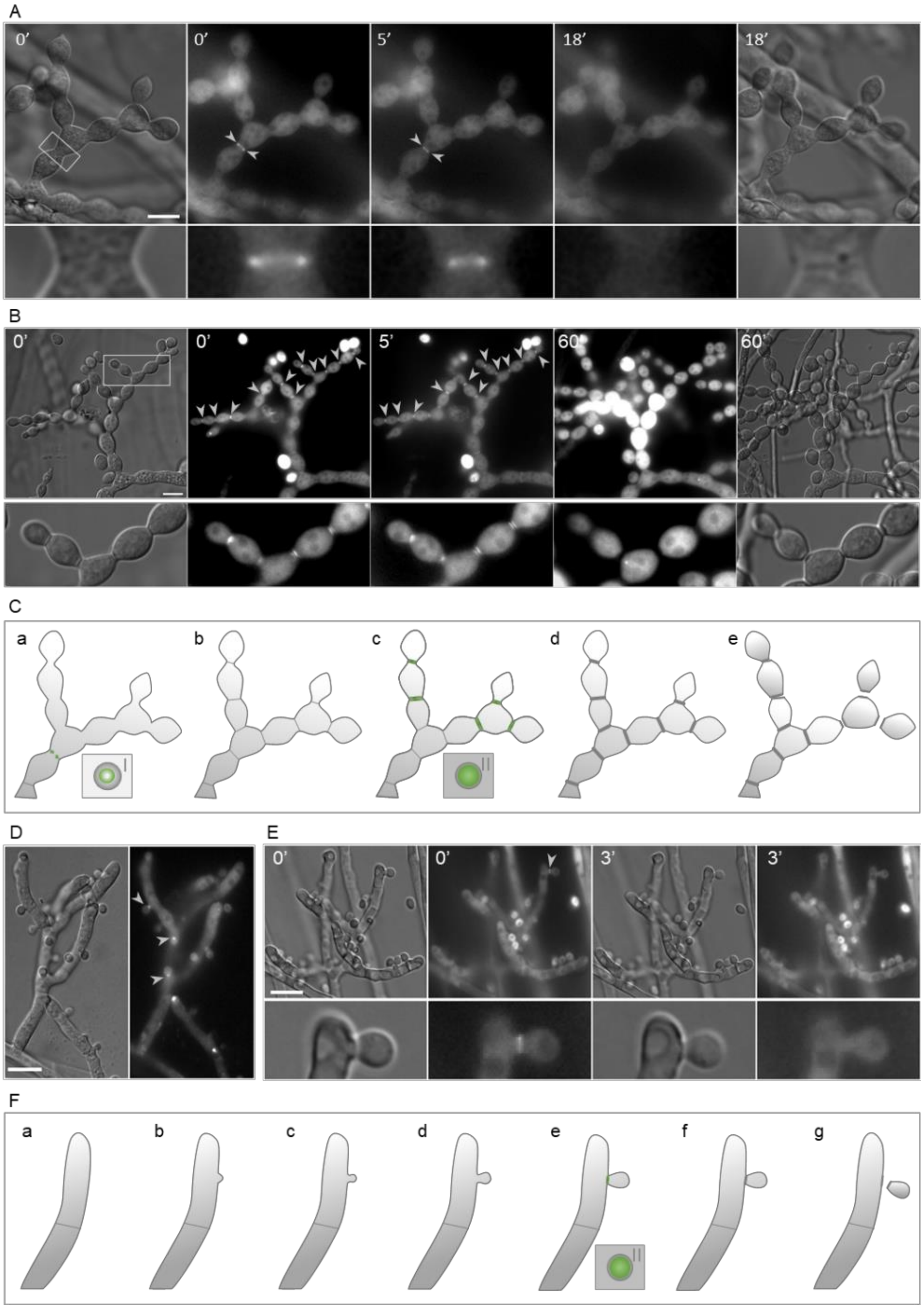


**S7 Fig: CSR-3 is recruited during vegetative conidiation.**

**(A/B)** Subcellular localization of GFP-CSR-3 (SH_125: *Pccg-1-gfp-csr-3*, Δ*csr-3*) (arrow heads) during the formation of macroconidia within an early (A) and a late (B) time point. **(C)** Illustration of the localization pattern of CSR-3 during the development of asexual macroconidia. **(D)** Localization of GFP-CSR-3 (arrow heads) during microconidiation. **(E)** CSR-3 is recruited to the conidiation site (0') and released (3') during microconidium formation **(F)** Illustration of the localization pattern of CSR-3 during the development of asexual microconidia. Strains were grown on plates with water agar. Scale bars = 10 µm. Time scale = minutes.
