# Supplementary material for: The chitin synthase regulator CSR-3 promotes cellular integrity during cell-cell fusion in the filamentous ascomycete fungus Neurospora crassa
Source: PLoS Genet. 2025 Oct 10;21(10):e1011891. doi: 10.1371/journal.pgen.1011891 (PMC12561907; doi:10.1371/journal.pgen.1011891)
Supplement: S8 Fig — (A,B) Recruitment of GFP-CSR-3 (SH_125: Pccg-1-gfp-csr-3, Δcsr-3) (arrow) to the burst tip (c) of a germling 2 minutes after addition of lysing enzyme (A) and SMC buffer as a control (B) (for corresponding movie see S2 Movie). (C,D) Localization of GFP-CSR-3 (SH_125: Pccg-1-gfp-csr-3, Δcsr-3) (arrow) at the tip of a germling 5 minutes after addition of tomatine (C) and DMSO as a control (D). (E) Quantification of CSR-3 localization patterns in response towards cell wall stress (lysing enzyme) and membrane stress (tomatine). Striped bars represent lysed cells (standard deviation values can be found in S4 Table). For details see material and method. Scale bars: 5 μm. Time scale: minutes. (PDF) [file pgen.1011891.s009.pdf]

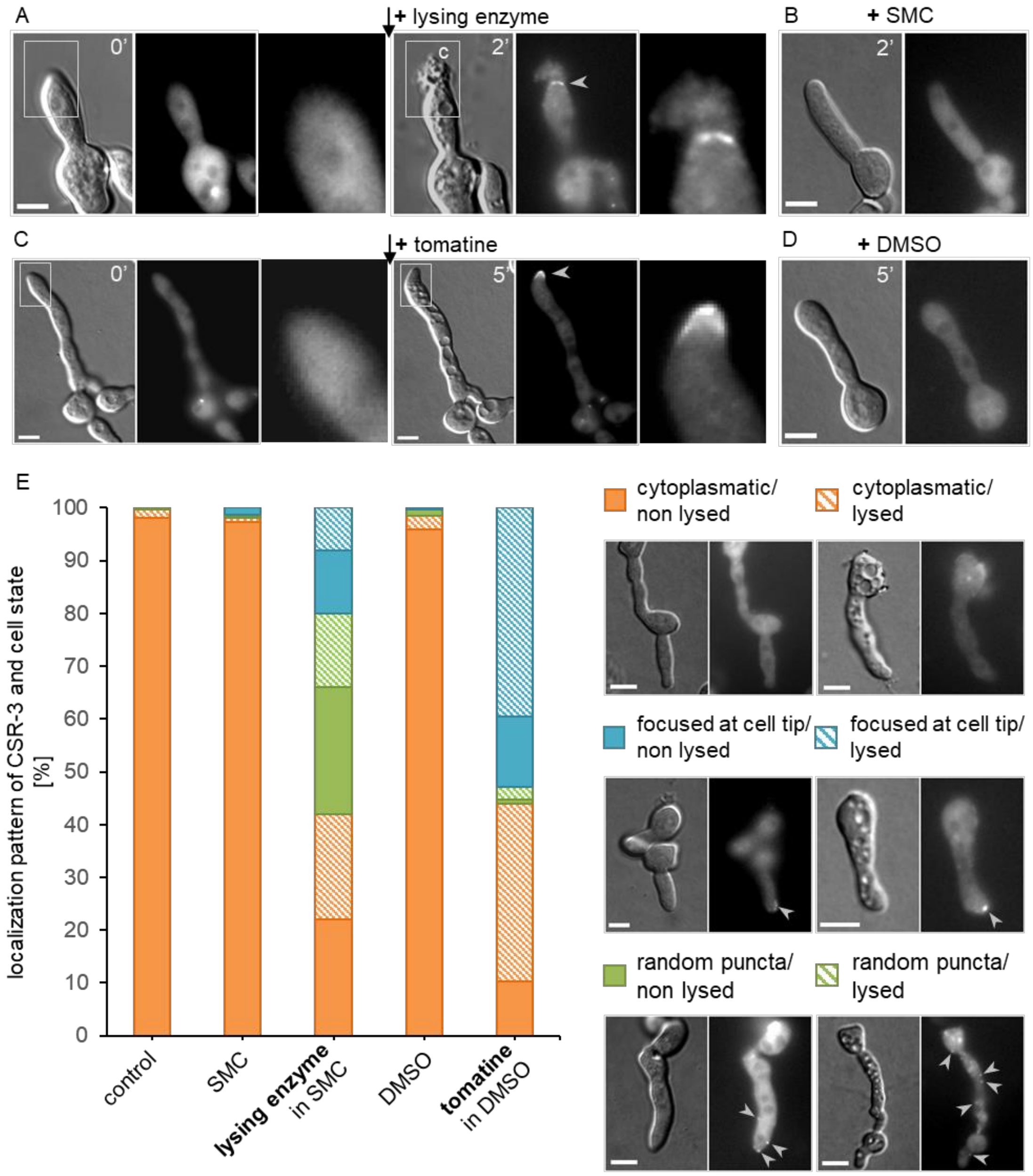

S8 Fig: CSR-3 localizes in at the cell periphery in response towards cell wall and membrane stress.

(A,B) Recruitment of GFP-CSR-3 (SH_125: *Pccg-1-gfp-csr-3*, Δ*csr-3*) (arrow) to the burst tip (c) of a germling 2 minutes after addition of lysing enzyme (A) and SMC buffer as a control (B) (for corresponding movie see **S2 movie**). **(C,D)** Localization of GFP-CSR-3 (SH_125: *Pccg-1-gfp-csr-3*, Δ*csr-3*) (arrow) at the tip of a germling 5 minutes after addition of tomatine (C) and DMSO as a control (D). **(E)** Quantification of CSR-3 localization patterns in response towards cell wall stress (lysing enzyme) and membrane stress (tomatine). Striped bars represent lysed cells (standard deviation values can be found in S4 table). For details see material and method. Scale bars: 5 µm. Time scale: minutes.
