# Supplementary material for: The chitin synthase regulator CSR-3 promotes cellular integrity during cell-cell fusion in the filamentous ascomycete fungus Neurospora crassa
Source: PLoS Genet. 2025 Oct 10;21(10):e1011891. doi: 10.1371/journal.pgen.1011891 (PMC12561907; doi:10.1371/journal.pgen.1011891)
Supplement: S9 Fig — 48 minutes time-lapse of subcellular localization of GFP-CSR-3 (SH_125: Pccg-1-gfp-csr-3, Δcsr-3) (arrow heads) after treatment with tomatine. Spores were cultivated in liquid MM, which is why they underwent a slight rearrangement after addition of the substance. For experimental details see material and method. Scale bars: 5 µm (overview) and 2 µm (inset). (PDF) [file pgen.1011891.s010.pdf]

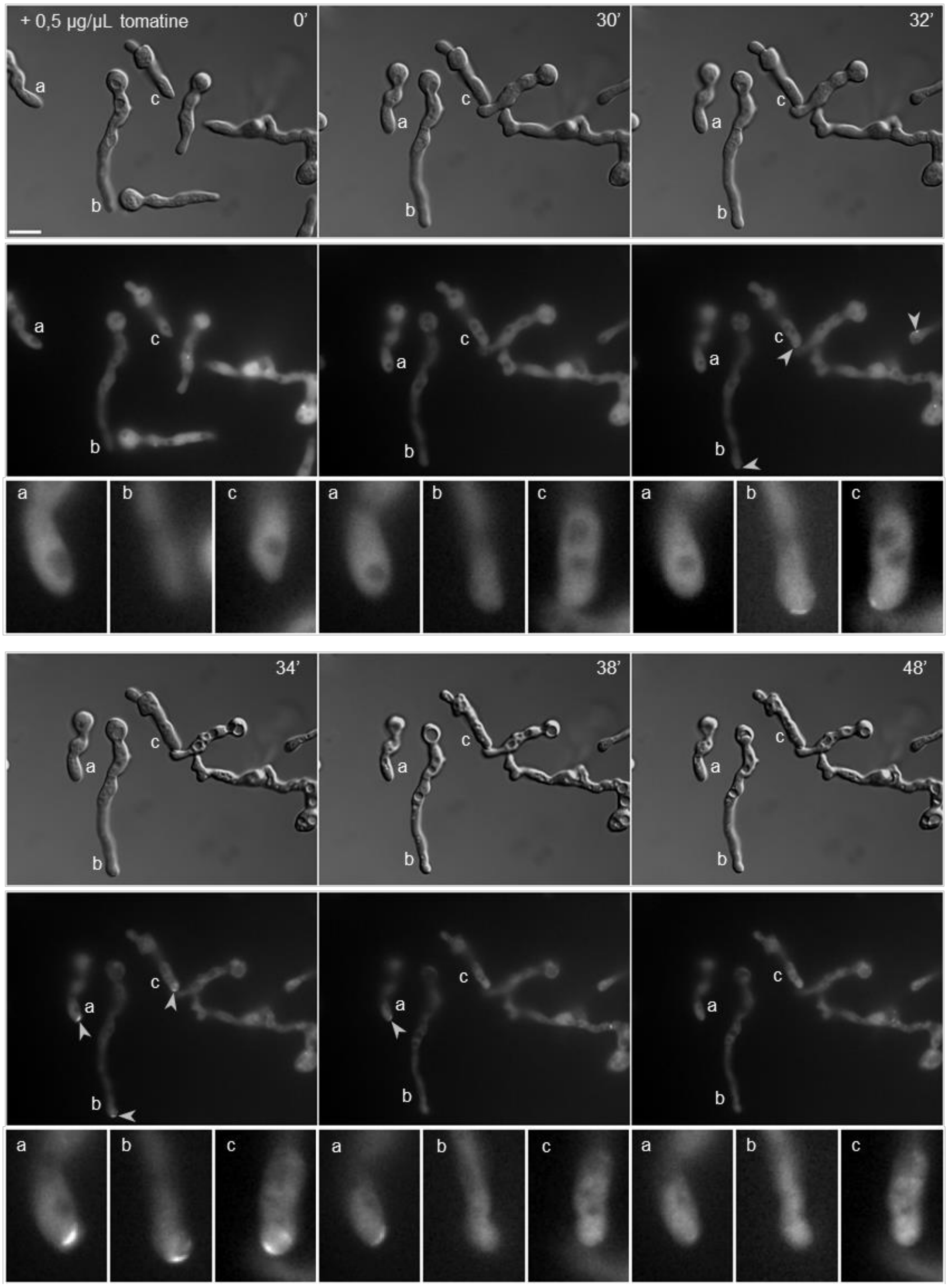

S9 Fig: CSR-3 is recruited dynamically to the tips of germlings treated with tomatine.

48 minutes time-lapse of subcellular localization of GFP-CSR-3 (SH_125: *Pccg-1-gfp-csr-3*, Δ*csr-3*) (arrow heads) after treatment with tomatine. Spores were cultivated in liquid MM, which is why they underwent a slight rearrangement after addition of the substance. For experimental details see material and method. Scale bar: 5µm.
