# Supplementary material for: The chitin synthase regulator CSR-3 promotes cellular integrity during cell-cell fusion in the filamentous ascomycete fungus Neurospora crassa
Source: PLoS Genet. 2025 Oct 10;21(10):e1011891. doi: 10.1371/journal.pgen.1011891 (PMC12561907; doi:10.1371/journal.pgen.1011891)
Supplement: S10 Fig — (A,B) 10-fold serial dilution of conidial suspensions of wild type (FGSC 2489) and the csr-3 deletion mutant (GN5-20) were spotted on BDES medium containing the relevant drug respectively or a drug free control. The growth differences were checked after incubation for 72 h and 120 h at 30 °C in darkness (for details see material and methods). (PDF) [file pgen.1011891.s011.pdf]

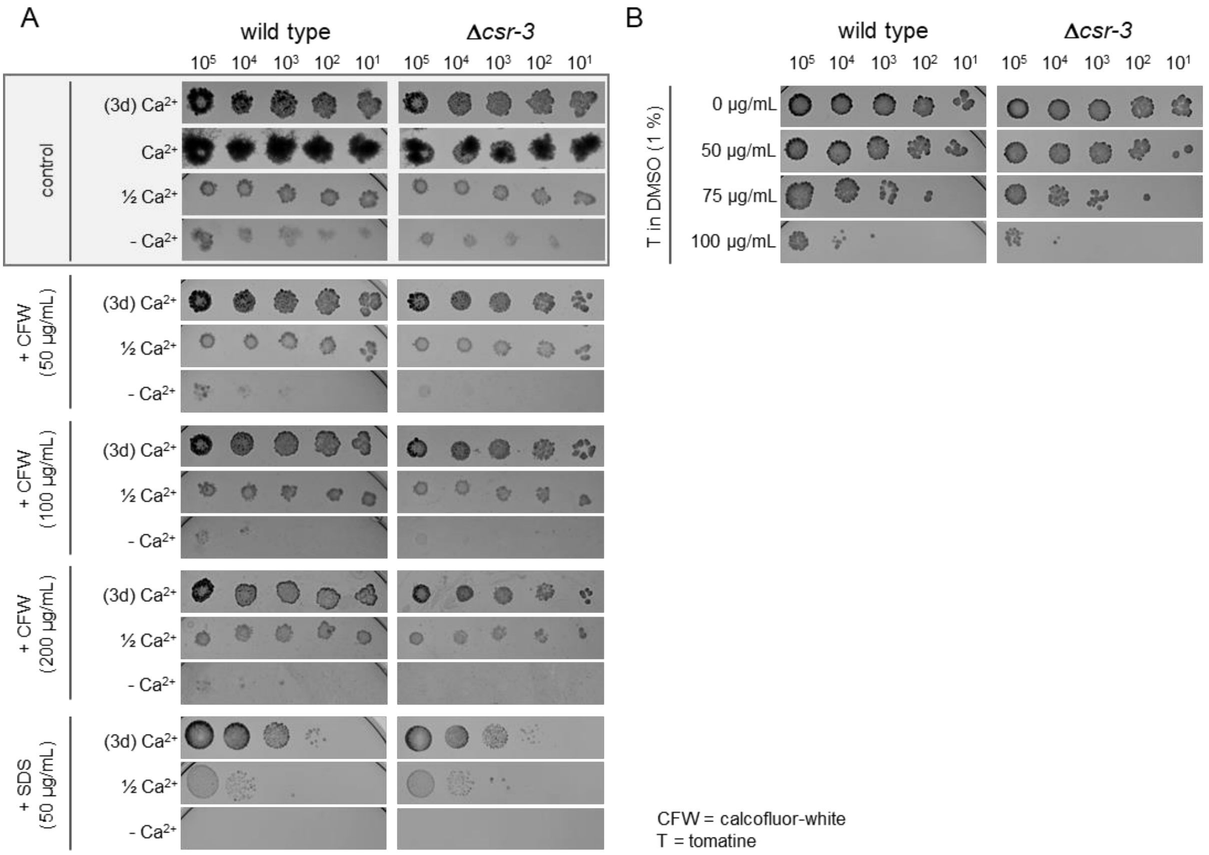

S10 Fig: Sensitivity-profile of *N. crassa* wt and Δ*csr-3* mutant to different membrane or cell wall attacking substances.

(A,B) 10-fold serial dilution of conidial suspensions of wild type (FGSC 2489) and the *csr*-3 deletion mutant (GN5-20) were spotted on BDES medium containing the relevant drug respectively or a drug free control. The growth differences were checked after incubation for 72 h and 120 h at 30 °C in darkness (for details see material and methods).
