# Supplementary material for: The chitin synthase regulator CSR-3 promotes cellular integrity during cell-cell fusion in the filamentous ascomycete fungus Neurospora crassa
Source: PLoS Genet. 2025 Oct 10;21(10):e1011891. doi: 10.1371/journal.pgen.1011891 (PMC12561907; doi:10.1371/journal.pgen.1011891)
Supplement: S11 Fig — (A) Co-cultivation of T. atroviride (CBS 122147) wild type strain with either N. crassa wild type (FGSC2489), Δcsr-3 mutant (GN5-20) or complemented mutant strain (SH_ 283: Pcsr-3-gfp-csr-3, Δcsr-3) on plates with MM after 4 days of incubation. (B) Quantification of the radius of the T. atroviride colony and the diameter of the lysed area within the N. crassa colony after 4 days of incubation. (C) Co-cultivation of T. atroviride (CBS 122147) wild type strain with either N. crassa wild type (FGSC2489), Δcsr-3 mutant (GN5-20) or complemented mutant strain (SH_ 283: Pcsr-3-gfp-csr-3, Δcsr-3) on plates with MM with only half calcium concentration after 4 days of incubation. (D) Quantification of the radius of the T. atroviride colony and the diameter of the lysed area within the N. crassa colony after 4 days of co-incubation of three independent samples. Statistically significant differences (p ≤ 0.05) are indicated by asterisks. For details of quantification, see materials and methods. (PDF) [file pgen.1011891.s012.pdf]

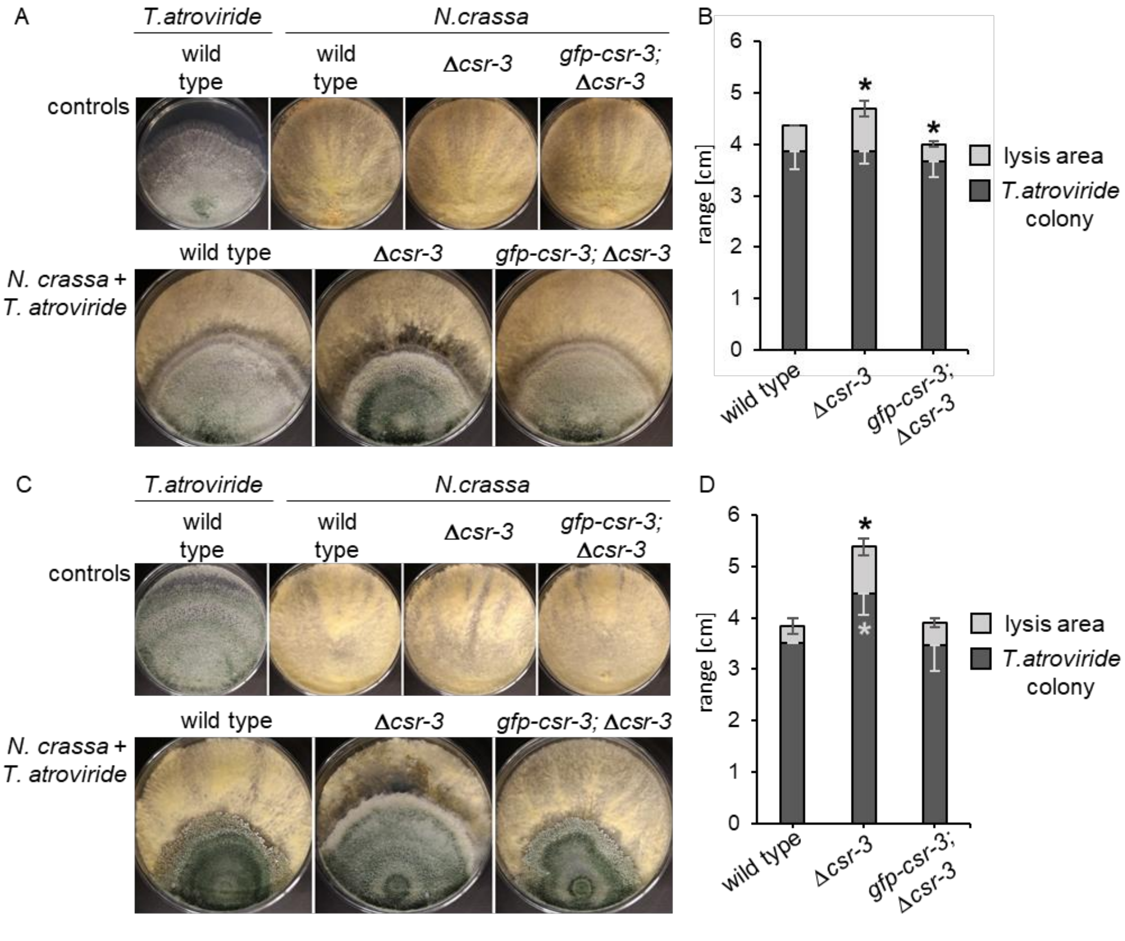

S11 Fig: CSR-3 promotes protection against the mycoparasite *T. atroviride*.

(A) Co-cultivation of *T. atroviride* (CBS 122147) wild type strain with either *N. crassa* wild type (FGSC2489), Δ*csr-3* mutant (GN5-20) or complemented mutant strain (SH_ 283: *Pcsr-3-gfp-csr-3*, Δ*csr-3*) on plates with MM after 4 days of incubation. **(B)** Quantification of the radius of the *T. atroviride* colony and the diameter of the lysed area within the *N. crassa* colony after 4 days of incubation. **(C)** Co-cultivation of *T. atroviride* (CBS 122147) wild type strain with either *N. crassa* wild type (FGSC2489), Δ*csr-3* mutant (GN5-20) or complemented mutant strain (SH_ 283: *Pcsr-3-gfp-csr-3*, Δ*csr-3*) on plates with MM with only half calcium concentration after 4 days of incubation. **(D)** Quantification of the radius of the *T. atroviride* colony and the diameter of the lysed area within the *N. crassa* colony after 4 days of co-incubation of three independent samples. Statistically significant differences ($p \leq 0.05$) are indicated by asterisks. For details of quantification, see materials and methods.
