# Supplementary material for: The chitin synthase regulator CSR-3 promotes cellular integrity during cell-cell fusion in the filamentous ascomycete fungus Neurospora crassa
Source: PLoS Genet. 2025 Oct 10;21(10):e1011891. doi: 10.1371/journal.pgen.1011891 (PMC12561907; doi:10.1371/journal.pgen.1011891)
Supplement: S12 Fig — (A) Localization of GFP-CSR-3 (SH_125: Pccg-1-gfp-csr-3, Δcsr-3) during fusion pore formation (arrow heads) in interacting germlings under treatment with 1-NM-PP-1. (B) Staining the cells with CFW revealed the successful formation of a fusion pore (fp) and septa (s). (C) Localization of MAK-1E104G-GFP during fusion pore formation (arrow heads) in interacting germlings (NCAL011-2: mak-1E104G-gfp, Δmak-1) under treatment with DMSO. (D) Staining the cells with CFW revealed the successful formation of a fusion pore (fp). (E,F) Addition of 1-NM-PP-1 prior to fusion results in the recruitment of MAK-1E104G-GFP in some pairs (E), but nevertheless pairs don’t succeed in fusion pore formation visible by remaining cell wall (arrow) and continues growth of germ tubes (F). For experimental details see materials and method. (PDF) [file pgen.1011891.s013.pdf]

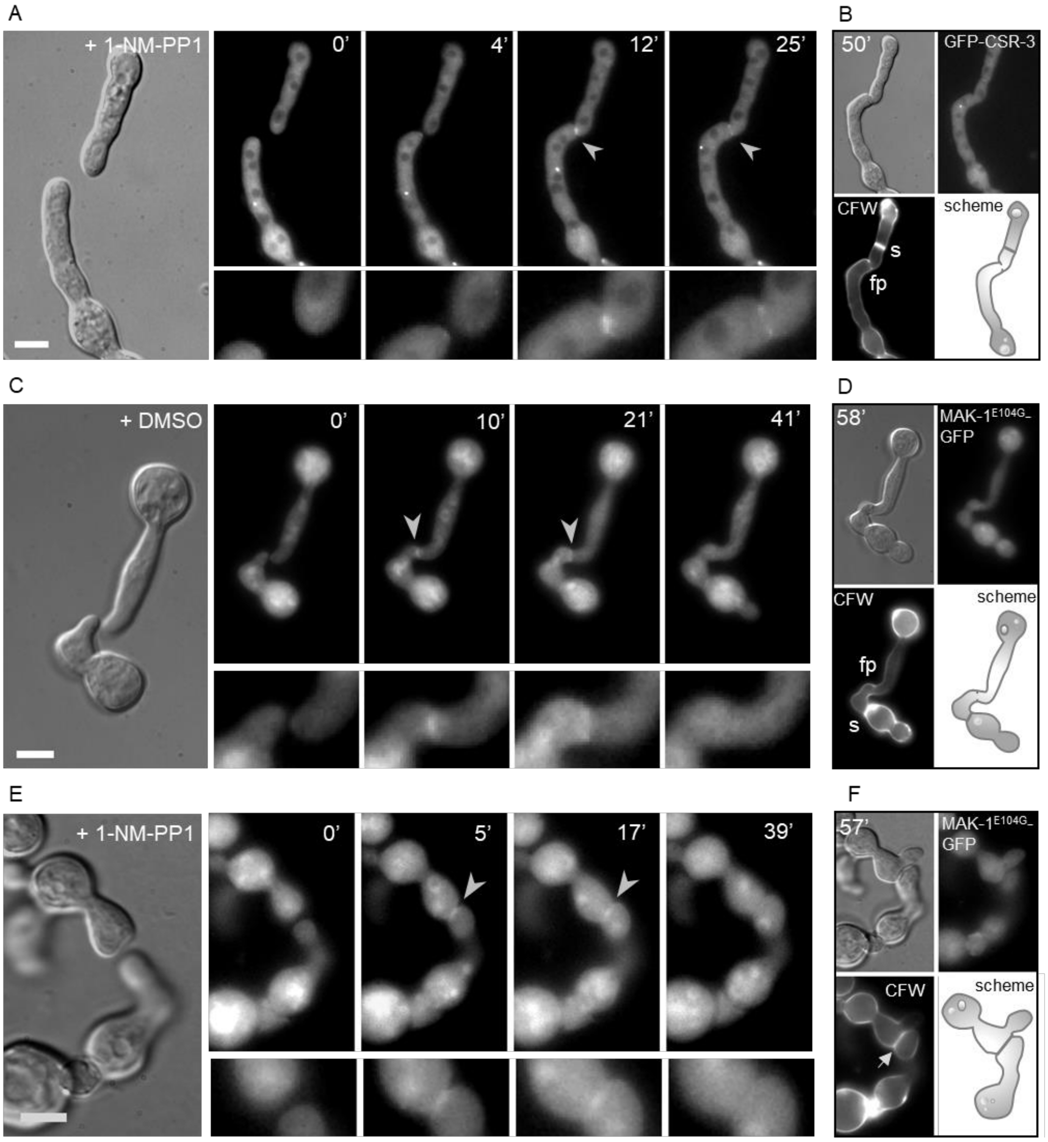

S12 Fig: Under treatment with 1-NM-PP-1 CSR-3's localization pattern is unaffected in a non-inhibitable background.

(A) Localization of GFP-CSR-3 (SH_125: *Pccg-1-gfp-csr-3*, Δ*csr-3*) during fusion pore formation (arrow heads) in interacting germlings under treatment with 1-NM-PP-1. **(B)** Staining the cells with CFW revealed the successful formation of a fusion pore (fp) and septa (s). **(C)** Localization of MAK-1^{E104G}-GFP during fusion pore formation (arrow heads) in interacting germlings (NCAL011-2: *mak-1*^{E104G}*-gfp*, Δ*mak-1*) under treatment with DMSO. **(D)** Staining the cells with CFW revealed the successful formation of a fusion pore (fp). **(E,F)** Addition of 1-NM-PP-1 prior to fusion results in the recruitment of MAK-1^{E104G}-GFP in some pairs (E), but nevertheless pairs don't succeed in fusion pore formation visible by remaining cell wall (arrow) and continues growth of germ tubes (F). For experimental details see materials and method.
