# Supplementary material for: The chitin synthase regulator CSR-3 promotes cellular integrity during cell-cell fusion in the filamentous ascomycete fungus Neurospora crassa
Source: PLoS Genet. 2025 Oct 10;21(10):e1011891. doi: 10.1371/journal.pgen.1011891 (PMC12561907; doi:10.1371/journal.pgen.1011891)
Supplement: S13 Fig — (A,B) Recruitment of GFP-CSR-3 (SH_297: Pccg-1-gfp-csr-3, Δchs-2) (arrow head) to the plug (p) of injured hyphae (A) and its corresponding quantification in comparison to the strain SH_125 (Pccg-1-gfp-csr-3, Δcsr-3) (B). Scale bar: 10 μm. (C) 3D-Z projection of a stack of 45 single images of GFP-CSR-3 during septum formation of strain SH_297 (Pccg-1-gfp-csr-3, Δchs-2). Scale bar: 3 μm. (D) Recruitment of GFP-CSR-3 (SH_297: Pccg-1-gfp-csr-3, Δchs-2) to the inner ring of a forming septum within a hypha. Membrane was stained with FM4-64. Scale bar: 2 µm. (E,F) Localization of GFP-CSR-3 (arrow heads) during germling fusion in a Δchs-2 mutant (SH_297: Pccg-1-gfp-csr-3, Δchs-2) (E) and its corresponding quantification in comparison to the strain SH_125 (Pccg-1-gfp-csr-3, Δcsr-3) (F). Scale bar: 5 µm. Error bars represent the standard deviation from at least three independent experiments (B: n = 15–25 hyphae each; F: n = 15–20 germlings each). Statistically significant differences (p ≤ 0.05) are indicated by asterisks. (PDF) [file pgen.1011891.s014.pdf]

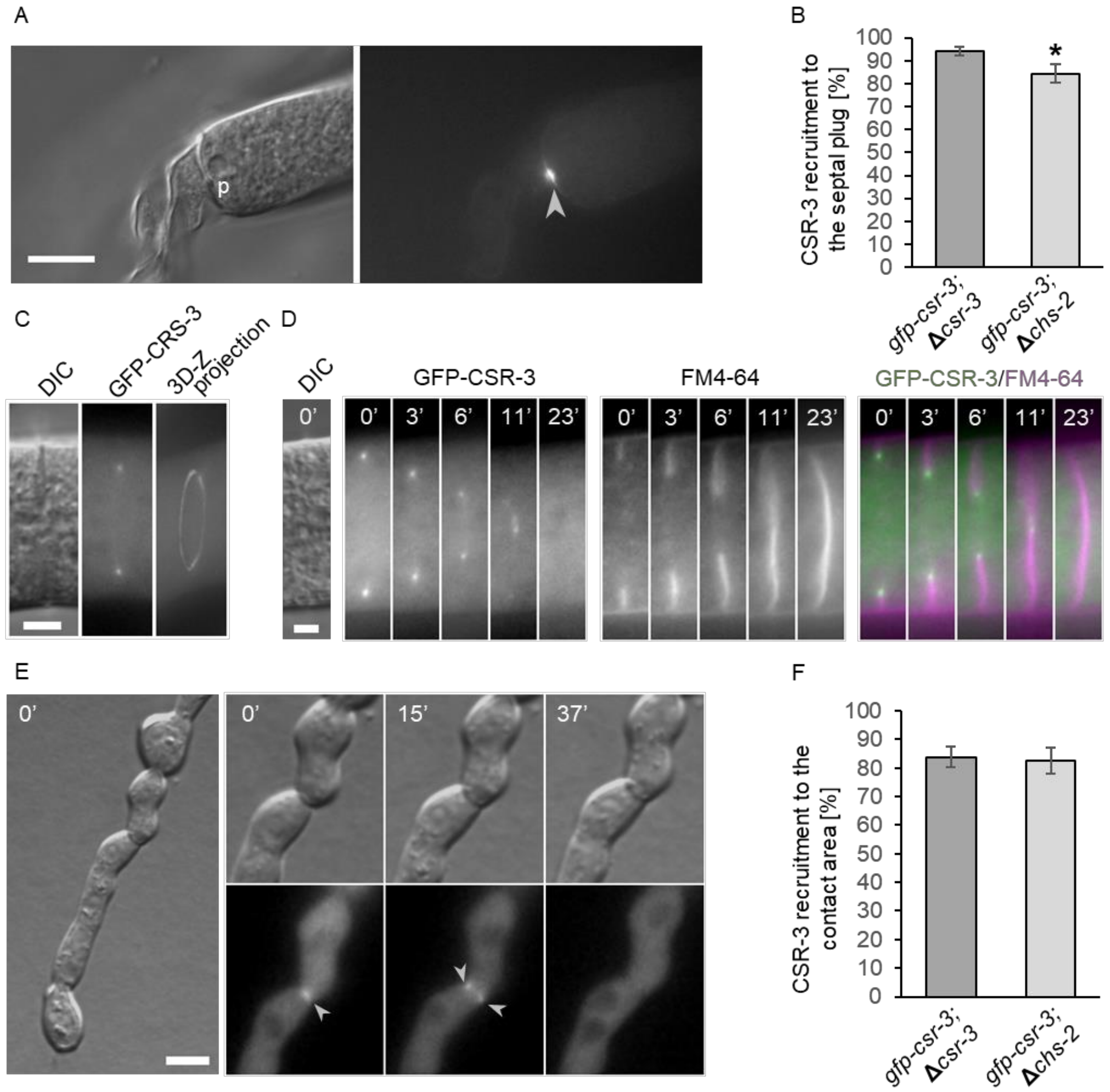

S13 Fig: CSR-3 localization pattern is not affected in a Δ*chs-2* mutant.

(A,B) Recruitment of GFP-CSR-3 (SH_297: *Pccg-1-gfp-csr-3*, Δ*chs-2*) (arrow head) to the plug (p) of injured hyphae (A) and its corresponding quantification in comparison to the strain SH_125 (*Pccg-1-gfp-csr-3*, Δ*csr-3*) (B). Scale bar: 10 µm. **(C)** 3D-Z projection of a stack of 45 single images of GFP-CSR-3 during septum formation of strain SH_297 (*Pccg-1-gfp-csr-3*, Δ*chs-2*). Scale bar: 3 µm. **(D)** Recruitment of GFP-CSR-3 (SH_297: *Pccg-1-gfp-csr-3*, Δ*chs-2*) to the inner ring of a forming septum within a hypha. Membrane was stained with FM4-64. Scale bar: 2 µm. **(E,F)** Localization of GFP-CSR-3 (arrow heads) during germling fusion in a Δ*chs-2* mutant (SH_297: *Pccg-1-gfp-csr-3*, Δ*chs-2*) (E) and its corresponding quantification in comparison to the strain SH_125 (*Pccg-1-gfp-csr-3*, Δ*csr-3*) (F). Scale bar: 5 µm. Error bars represent the standard deviation from at least three independent experiments (B: n = 15-25 hyphae each; F: n= 15-20 germlings each). Statistically significant differences ($p \leq 0.05$) are indicated by asterisks.
