# Supplementary material for: The chitin synthase regulator CSR-3 promotes cellular integrity during cell-cell fusion in the filamentous ascomycete fungus Neurospora crassa
Source: PLoS Genet. 2025 Oct 10;21(10):e1011891. doi: 10.1371/journal.pgen.1011891 (PMC12561907; doi:10.1371/journal.pgen.1011891)
Supplement: S1 Table — (PDF) [file pgen.1011891.s015.pdf]

S1 Table. Strains used in this study

strain	genotype	origin
Trichoderma atroviride		
GT-13	CBS 122147 Trichoderma atroviride WT	Westerdijk institute
Neurospora crassa		
FGSC 2489	*mat A*	FGSC
FGSC 988	*mat a*	FGSC
FGSC 4564	*a^{m1}*, *ad-3B*, *cyh-1*	FGSC
FGSC 6103	*his-3^{-}*, *mat A*	FGSC
FGSC 9719	Δ*mus-52*::*bar*, *mat a*	FGSC
FGSC 22804	Δ*chs-2*::*hph*, *mat A*	FGSC
SMRP285	P*chs-2*::*chs-2*::*sgfp* Δ*mus-51*::*bar^{+}*, *mat a*	(FAJARDO-SOMERA *et al.* 2015)
SMRP286	P*chs-4*::*chs-4*::*sgfp* Δ*mus-51*::*bar^{+}*,*mat a*	(FAJARDO-SOMERA *et al.* 2015)
SMRP287	P*chs-5*::*chs-5*::*sgfp* Δ*mus-51*::*bar^{+}*,*mat a*	(FAJARDO-SOMERA *et al.* 2015)
SMRP288	P*chs-7*::*chs-7*::sgfp Δ*mus-51*::*bar^{+}*, *mat a*	(FAJARDO-SOMERA *et al.* 2015)
SMRP30	*his3^{+}*::P*ccg-1-chs-6^{+}-sgfp^{+}*,*mat A*	(RIQUELME *et al.* 2007)
SMRP31	*his3^{+}*::P*ccg-1-chs-3^{+}-sgfp^{+}*, *mat A*	(RIQUELME *et al.* 2007)
SMRP90	P*ccg-1-chs-1-ChFP, mat A*	(VERDIN *et al.* 2009)
N1-22	*his3^{+}*::P*ccg1-so-gfp*, *mat A*	(FLEISSNER AND GLASS 2007)
N3-06	*his-3^{+}*::P*ccg-1-gfp*, *mat A*	(FLEISSNER *et al.* 2009)
N3-07	*his-3^{+}*::P*ccg-1-mCherry*, *mat A*	(SCHÜRG *et al.* 2012)
GN4-32	Δ*mak-1*::*hph, his-3^{+}*::P*ccg-1-gfp-mak-1,* mat A	(DETTMANN *et al.* 2013)
GN5-20	Δ*csr3*::*hph*, *mat A*	this study
GN5-21	Δ*csr3*::*hph*, *mat a*	this study
NCAL011-2	Δ*mak-1*::*hph*,P*ccg-1-mak-1^{E104G}-gfp* (EC), *mat A*	(WEICHERT *et al.* 2016)
MW_581	Δ*mek-1*::*hph, his-3^{+}*::P*ccg-1-mek-1-gfp*	Martin Weichert, TU Braunschweig
AS-111-848	Δ*mak-1*::*hph*-P*ccg-1-mak-1^{E104G}*, his-3^{-}, *mat A*	Antonio Serrano, TU Braunschweig
SH_1	Δ*csr-3*::*hph, his-3^{-}*, *mat A*	this study
SH_9-11	Δ*csr-3*::*hph, his-3^{+}*::*Pccg-1-mCherry*, *mat A*	this study
SH_12-14	Δ*csr-3*::*hph, his-3^{+}*::P*ccg-1-gfp*, *mat A*	this study
SH_45	*his-3^{+}*::P*ccg-1-gfp-csr-3, mat A*	this study

SH_59	Δ*csr-3*::*hph, his-3*$^{+}$::P*ccg-1-gfp-csr-3*, *mat A*	this study
SH_94/95	Δ*csr-3*::*hph, his-3*$^{+}$::P*ccg-1-dsRED-csr-3*, *mat A*	this study
SH_125	Δ*csr-3*::*hph his-3*$^{+}$::P*ccg-1-gfp-csr-3*, *mat A*	this study
SH_126	Δ*csr-3*::*hph his-3*$^{+}$::P*ccg-1-gfp-csr-3*, *mat a*	this study
SH_146	Δ*chs-2*::*hph* Δ*csr-3*::*hph, mat A*	this study
SH_157	*his-3*$^{+}$::P*ccg-1-chs-2-gfp*, *mat A*	this study
SH_161	Δ*chs-2*::*hph, his-3*$^{+}$::P*ccg-1-chs-2-gfp,mat a*	this study
SH_171	*Pcsr-3-gfp-csr-3*^{VAA}, Δ*csr-3*::*hph*	this study
SH_176	*Pcsr-3-gfp-csr-3*^{EDD}, Δ*csr-3*::*hph*	this study
SH_230	Δ*mak-1::mak-1*^{E104G}*-hph,* Δ*csr-3::hph, his-3*$^{+}$::P*ccg-1-gfp-csr-3, mat A*	this study
SH_248	*his-3*$^{+}$::P*ccg-1-gfp-csa2, mat A*	this study
SH_250	*his-3*$^{+}$::P*ccg-1-gfp-csa1, mat A*	this study
SH_257	Δ*csr-3*::*hph his-3*$^{+}$*::* P*ccg-1-csr-3-gfp*, *mat A*	this study
SH_261	Δ*mik-1*::*hph his-3*$^{+}$::P*ccg-1-gfp-8xgly-mik-1, mat A*	this study
SH_283	Δ*csr-3*::*hph his-3*$^{+}$::P*csr-3-gfp-csr-3*, *mat A*	this study
SH_290	Δ*csr-3*::*hph his-3*$^{+}$::P*csr-3-gfp-csr-3*^{C932S}*(SAAX)*, *mat A*	this study
SH_310	*Pccg-1-gfp-csr-3*^{VAA}, Δ*csr-3*::*hph*	this study
SH_314	*Pccg-1-gfp-csr-3*^{EDD}, Δ*csr-3*::*hph*	this study
SH_316	Δ*csr-3*::*hph, his-3*$^{+}$::P*ccg-1-gfp-csr-3*^{C932S}, *mat A*	this study
NCAL011-2	Δ*mak-1*::*hph, mak-1(E104G)-gfp-bar* random integration (phenotypic rescue), *mat A*	Alexander Lichius, University of Edinburgh
