# Supplementary material for: The chitin synthase regulator CSR-3 promotes cellular integrity during cell-cell fusion in the filamentous ascomycete fungus Neurospora crassa
Source: PLoS Genet. 2025 Oct 10;21(10):e1011891. doi: 10.1371/journal.pgen.1011891 (PMC12561907; doi:10.1371/journal.pgen.1011891)
Supplement: S2 Table — (PDF) [file pgen.1011891.s016.pdf]

S2 Table. Oligonucleotides used in this study

No.	Nucleotide sequence (5´–3´)
82	GTCGGAGACAGAAGATGATATTGAAGGAGC
83	GTTGGAGATTTCAGTAACGTTAAGTGGAT
629	GTAACGCCAGGGTTTTCCCAGTCACGACGATGTAGATACTCCGCCTTGG
630	ATCCACTTAACGTTACTGAAATCTCCAACAAGAGAGAAAGGGAGAGAGG
631	CTCCTTCAATATCATCTTCTGTCTCCGACACGCTATGTGCAGTAGAAGG
632	GCGGATAACAATTTCACACAGGAAACAGCTCAACCTCTGGAGTACCTGG
644	GATTATTATCTAGAATGCCTACCTTGGGCGCAACGTTTGTCC
645	ATATCCTATTAATTAACATTACAACCTATTTGTAACCC
936	AATGTAagatctATGCCTACCTTGGGCGCAAC
937	AATGTAtctagaGACCTCCGAAGCAACCCGCG
1074	AATGTAGGGCCCGGCTCTTCTCCAGTTGTGAG
1075	AATGTAGAATTCTTCGGGGGAGTCTTTTTCTG
1219	GTAACGCCAGGGTTTTCCCAGTCACGACGagatctATGCCTACCTTGGGCGCAAC
1221	CTGTCGTAGGCACCGATGGCCGT
1224	ACGGCCATCGGTGCCTACGACAG
1229	GCGGATAACAATTTCACACAGGAAACAGCtctagaGACCTCCGAAGCAACCCGCG
1231	CAGACAACCCGCCCCCGGACCTGGCCCCGGCCCTGGCGCCGGCCCTCGAC
1233	CTGTCGTAGGCTCCGATGGCCGT
1234	ACGGCCATCGGAGCCTACGACAG
1236	GTCGAGGGCCGTCGCCAGGGCCGGGGCCAGGTCCGGGATCGGGTTGTCTG
1239	CAGACAACCCGATCCCGGACCTGGCCCCGGCCCTGGCGACGGCCCTCGAC
1523	GTAACGCCAGGGTTTTCCCAGTCACGACGgggcccTCTTCTCCAGTTGTGAGC
1525	ACGACAGCGACaGCGTAAGTTTACTTCTCCT
