# Supplementary material for: The chitin synthase regulator CSR-3 promotes cellular integrity during cell-cell fusion in the filamentous ascomycete fungus Neurospora crassa
Source: PLoS Genet. 2025 Oct 10;21(10):e1011891. doi: 10.1371/journal.pgen.1011891 (PMC12561907; doi:10.1371/journal.pgen.1011891)
Supplement: S3 Table — (PDF) [file pgen.1011891.s017.pdf]

S3 Table. Plasmids used in this study

No.	Name	Features/Purpose	Origin
18	pMF334-GFP	n-terminal GFP tag, integration at *his-3* locus	Elizabeth Hutchison, UC Berkeley
10	pMFcherry	*Pccg-1-mCherry*, integration at *his-3* locus	(SCHÜRG *et al.* 2012)
2	pMF272	*Pccg-1-sgfp*, integration at *his-3* locus	(FREITAG *et al.* 2004)
11	pCSN44	*hph* resistance gene	(COLOT *et al.* 2006)
530	GFP-CSR-3	CSR-3 with n-terminal tagged GFP	this study
559	dsRED-CSR-3	CSR-3 with n-terminal tagged dsRED	this study
362	CSR-3-GFP	CSR-3 with c-terminal tagged GFP	this study
587	Pcsr-3-GFP-CSR-3	CSR-3 with n-terminal tagged GFP under control of the native promotor	this study
838	Pccg-1-GFP-CSR-3^{SAAX}	CSR-3 with SaaX instead of CaaX motif with n-terminal tagged GFP	this study
814	Pcsr-3-GFP-CSR-3^{SAAX}	CSR-3 with SaaX instead of CaaX motif with n-terminal tagged GFP, under control of the native promotor	this study
776	Pccg-1-GFP-CSR-3^{VAA}	CSR-3 mutated phophosites with n-terminal tagged GFP	this study
783	Pccg-1-GFP-CSR-3^{EDD}	CSR-3 mutated phophosites with n-terminal tagged GFP	this study
836	Pcsr-3-GFP-CSR-3^{EDD}	CSR-3 mutated phophosites with n-terminal tagged GFP, under control of the native promotor	this study
837	Pcsr-3-GFP-CSR-3^{VAA}	CSR-3 mutated phophosites with n-terminal tagged GFP, under control of the native promotor	this study
