# Supplementary material for: The chitin synthase regulator CSR-3 promotes cellular integrity during cell-cell fusion in the filamentous ascomycete fungus Neurospora crassa
Source: PLoS Genet. 2025 Oct 10;21(10):e1011891. doi: 10.1371/journal.pgen.1011891 (PMC12561907; doi:10.1371/journal.pgen.1011891)
Supplement: S4 Table — (PDF) [file pgen.1011891.s018.pdf]

S4 Table. Standard Deviations for figure S8

	cytoplasmatic signal		random puncta signal		focused signal at the tip	
	non lysed	lysed	non lysed	lysed	non lysed	lysed
**control**						
mean value (%)	99,63	1,47	0,37	0,37	0,00	0,00
STDEV (%)	0,64	1,70	0,64	0,64	0,00	0,00
**SMC**						
mean value (%)	97,98	0,62	0,69	0,33	1,33	0,00
STDEV (%)	1,02	1,08	0,60	0,57	0,57	0,00
**5% DSMO**						
mean value (%)	98,48	2,55	1,19	0,00	0,33	0,00
STDEV (%)	1,85	2,79	2,06	0,00	0,57	0,00
**lysis enzyme (30 µg/µL) in SMC**						
mean value (%)	42,00	20,00	38,00	14,00	20,00	8,00
STDEV (%)	10,82	12,12	4,58	11,36	4,58	4,58
**tomatine (0,5 µg/µL) in DMSO (5%)**						
mean value (%)	44,01	33,64	3,13	2,43	52,86	39,61
STDEV (%)	12,11	19,07	4,61	3,40	12,21	9,40
