# Supplementary material for: The chitin synthase regulator CSR-3 promotes cellular integrity during cell-cell fusion in the filamentous ascomycete fungus Neurospora crassa
Source: PLoS Genet. 2025 Oct 10;21(10):e1011891. doi: 10.1371/journal.pgen.1011891 (PMC12561907; doi:10.1371/journal.pgen.1011891)
Supplement: Attachment — Submitted filename: Response to Reviewers.pdf [file pgen.1011891.s021.pdf]

Dear Michael,

We sincerely thank you and the reviewers for the thoughtful and constructive feedback on our manuscript. We are encouraged by the positive reception and appreciative remarks. We have addressed all points raised, agreeing with nearly all of them, and we believe this input has substantially improved the manuscript. Below, we provide our point-by-point responses to the reviewers' comments.

Editor:

***First**, I agree with Reviewer 2 that some of the structures shown look like CATs; please comment on plating density of spores and whether you can ascertain that these are CATs or "standard" germ tubes.*

This point touches on the long-standing debate of whether *N. crassa* germlings fuse via germ tubes, CATs, or both. The term CAT was introduced based on analyses predominantly performed in liquid culture, where fusion often involves structures distinct from germ tubes. In contrast, our experiments are largely conducted on solid media, where most fusion events involve germ tube tips. Importantly, beyond terminology and minor morphological differences, no functional distinction has been identified between fusing CATs and fusing germ tube tips. All protein localization patterns examined so far support this view. It is therefore often impossible to distinguish if a fusing tip is a CAT, a germ tube, or a germ tube which has differentiated into a CAT. Therefore, we regard this debate as largely semantic and have chosen not to engage with it in detail here, as doing so in each of our papers would require an extensive discussion without advancing the biological insights. For this reason, we consciously avoid revisiting the issue in the present manuscript and hope that this is acceptable. However, we did include information on plating density to the material and methods section.

***Second**, I agree that when writing about chitin synthases one should be explicit because the naming conventions change from organism to organism.*
We agree that this is an important point and have now included the chitin synthase class when referring to these proteins in the manuscript.

***Third**, though many folks claim that genes driven by the ccg-1/grg-1 promoter are overexpressed, unless you have data to that effect (by RT=PCR or northern) it is difficult to ascertain this. While the grg-1/ccg-1 gene is highly expressed in aerial hyphae and spores it tends to resemble a housekeeping gene in liquid and high glucose medium.*
We agree with this valid point. Since we did not compare expression strength in this study, we removed the term 'overexpression' and now state that this promoter is routinely used for protein localization studies, citing the original publication in which it was established for this purpose.

***Lastly**, while folks refer to N. crassa as a "bread mold" this statement can be dropped without harm, especially as we don't even seem to be able to agree on the color ("red", "orange", and I know it as the "pink" bread mold). The name was originally given by folks who were looking at N. sitophila, so perhaps just call it Neurospora?*
We agree and have removed this statement from the manuscript, and we will avoid using it in future work.

Reviewer #1*:*
This is a very well written manuscript describing a thorough investigation on the role of CSR-3 in cell wall remodeling events. There are only very minor changes that this reviewer recommends.
In referring to the prenylation signal the manuscript uses CAAX and CaaX designations. Since the a's refer to hydrophobic amino acids, I think the authors mention to use Caax throughout the manuscript.

We changed the term to Caax throughout the manuscript

The authors will want to double-check spellings - I think the phosphoproteom on line 350 should be phosphoproteome. These are very minor issues and the reviewer comments the authors on an excellent study and manuscript.

We double-checked the manuscript and corrected all typos we found.

Reviewer #2:

The article “The chitin synthase regulator CSR-3 promotes cellular integrity during cell-cell fusion in the filamentous ascomycete fungus Neurospora crassa” by Herzog et al., is very well-written and presents a substantial amount of high-quality data aimed to discern the localization and role of the chitin synthase regulator CSR-3 during several developmental stages in N. crassa. The article provides valuable information in an area of cell wall fungal biology that is understudied. While all the experiments are conducted carefully and results are presented very elegantly, several points require clarification or revision to improve precision and accessibility for a broader scientific audience:

- The authors refer to Neurospora crassa as the red bread mold (Page 3, Line 50). It is more widely referred in the literature to as the orange bread mold.

We deleted this term entirely and only use the species name

- Page 3, line 58: The text should read CHS-2

has been fixed

- Page 4, lines 77-78: The wording is a bit confusing. What do the authors mean by “not only mating fusion”? And by “identical cells at two distinct developmental stages”?

We edited the wording and hope that the sentence is now better to understand.

- Page 4, line 81: What does the phrase “hyphal branches fuse to create cross connections” mean? Unclear what the cross connections refer to.

we added "between leading hyphae" to explain what the cross connections are connecting

- Page 5, line 94: Referring to th cell wall as an organelle is inaccurate, as organelles are typically membrane-bound. It would be more appropriate to describe it as a structure.

We agree, this has been fixed

- The nomenclature for chitin synthases is inconsistent across fungal taxa. It would be helpful if the authors specified the class of chitin synthases when referring to a specific CHS?

We added information on the respective class, when we mention the CHS for the first time

- Page 6, line 119: Why do the authors say chitin synthases contribute to cell wall remodeling? What about biosynthesis?

we changed the wording to "synthesis and remodeling"

- S2A Fig: Unclear why all transformants exhibit a signal at 4100 bp, apparently because of the mus52 deletion in strain FGSC9719. Please provide a brief explanation.

We added this information to the figure legend. It is indeed caused by hybridization of the protrpC promoter in the probe and the resistance marker integrated at the mus52 locus

- Page 8, line 168: Please add "in the csr-3 knockout strains" after "were unaffected"

has been fixed

- Page 9, lines 206-208: Under which conditions was the Pccg-1used to overexpress constructs?

the issue of overexpression has been fixed and strains were grown under standard conditions as described in the Material and Methods section

- Fig. 2B: Is the structure shown a CAT (conidial anastomosis tube) rather than a germ tube? There are similar structures throughout the manuscript (Fig. 7 B and C for instance) that appear to be CATS. There is no mention of this in the article. Please clarify and discuss the significance.

Please see our comment to the editor.

- Fig. 2D, E: What is the nature of the expanding ring-like structure shown? This is commonly seen in N. crassa. Please elaborate.

We do state in the manuscript that this structure represents the forming fusion pore, the sentence reads: "In time lapse, the signal appeared as an expanding ring, suggesting that CSR-3 localizes around the forming fusion pore"
We are not certain what is meant by "this is commonly seen in *Neurospora*." While it is true that other proteins involved in fusion display similar patterns, the precise structure, architecture, and sequence of events remain unclear. We therefore believe that further elaboration on this point would be highly speculative and prefer to refrain from doing so.

- Several figures are missing scale bars, particularly in magnified insets (For example, Figs. 2, 3, 4, 5B, E, 6, 7, 8, 9, and S8, S9, S12 Figs. etc.). Please ensure all images include scale information.

We included scale bars in all images

- Fig. 6A: Please indicate what the sample was stained with.

We included the information that the membranes were stained with FM4-64

- Page 11, line 246: Was the observed hyphal injury natural or experimentally induced? Please specify.

We included the information that the injury was induced by razor blade cutting

- Page 11, line 252: Please provide a reference for microconidia formation.

We added such a reference

- Page 12, lines 274-275: In which figure is GFP-CSR-3 seen accumulating at the cell periphery?

We added the information that this localization is seen in S8E Fig. and added to the figure the term "at the cell periphery"

- S8 Fig: The term should be "cytoplasmic", not "cytoplasmatic"

This has been fixed

- Page 13, line 294: "Therefor" should be "Therefore"

This has been fixed

- Page 13, lines 301-304: Please rewrite for better clarity.

We changed the wording for better readability.

- Page 14, line 312: Please rewrite to improve readability.

We changed the wording for better readability.

- Page 14, line 316: S11 Fig, should be S11 A,B Fig; line 318 add (S11 C, D Fig) after calcium concentration.

This has been fixed

- Page 15, line 350: Correct “phosphoproteom” to “phosphoproteome”

This has been fixed

- Revise Fig. 7 legend. All panels A, B, and C mention constructs based on mik-1, whereas panel B appears to be based on mek-1, and panel C on mak-1. Please correct this for accuracy.

This has been fixed

- Is it GFP-MAK-1 or MAK-1-GFP? Figure 7 and Figure 8 show different versions. Interestingly, in both figures MAK-1 tagged with GFP is also observed at what looks like nuclei. The authors do not mention this. Could it be relevant? Also, in figure 8, dsRed-CSR-3 appears at some globular organelles, not observed when imaging the GFP version. What are these organelles? Could the authors comment on this?

This was a labeling mistake and has been fixed (always GFP-MAK-1). The localization pattern to nuclei has been described in an earlier study and we see no relevance for this manuscript (or the function and regulation of CSR-3). Similarly, the dsRED signal in globular organelles is always seen when using this fluorescent protein (at least in our hands). We believe that it is accumulating in vacuoles as part of protein degradation. However, since this is only a speculation, we would prefer not to comment on it.

- MAK-1 and CSR-3, or SO and CSR-3 do not seem to co-localize during the entire duration of fusion pore formation based only on the visual evaluation of Figure 8. In some panels there seems to be signal of one protein but not the other, indicative of sequential appearance. Could the authors do a quantitative co-localization analysis using image analysis software to have more informative data? It would strengthen the observations.

We already tried to do a more detailed sequential analysis, however, the data were somewhat inconclusive. we agree that this would be very valuable information, however we were not able to provide absolutely conclusive data. With this said, we adjusted to statement deleting the statement "and were synchronously released when fusion was completed", since this statement was indeed not fully supported. We very much thank the reviewer for pointing out this lapsus!

- In Figure 9 legend: For Pccg-1-gfp-csr-3, mak- 1E104G, Δcsr-3, Δmak-1 and so on, could the authors organize the genotypes so that it is clearer for readers which deletion strain was complemented by which construct? What are the round organelles depicted in the schemes? Why some drawn but not others?

We have updated the labeling to clearly indicate the expression constructs and the genotype of the recipient strain. The round organelles, which were purely decorative and irrelevant, have been removed. Thank you for pointing this out.

- Page 18, lines 425-426: This sentence is unclear. Do the authors mean that localization of CSR-3 did not require CHS-2? Please rephrase and elaborate on the reasoning.

We edited the wording accordingly

- Page 19, first paragraph: What was the lysis rate on the mutant under normal calcium conditions?

We added additional data showing the lysis rates of the various strains under normal calcium conditions to Fig. 10D

- The supplementary movies play too quickly, making it difficult to follow events such as cytoplasmic leakage or fluorescence changes. Could the authors provide versions that run slower?

Unfortunately, we were unable to resolve this issue. However, when downloading movies from PLOS Genetics articles, we can adjust the playback speed in our media players. We therefore expect that readers of our publication will also be able to control the speed while viewing our movies. We confirmed this with our movies in Windows Media Player, where speed adjustment was also possible.

- Discussion:
- page 20
o Line 468: The fusion focus hypothesis? What do you mean?

The description of this hypothesis appears immediately before this sentence. To clarify the reference, we changed "the hypothesis" to "this hypothesis."

o Lines 471-474. The authors state that prior to their studies no cell wall remodeling enzyme mediating cell fusion in filamentous fungi had been identified. Check Martinez-Núñez and Riquelme, 2015. Fungal Genetics and Biology. http://dx.doi.org/10.1016/j.fgb.2015.11.001. There the authors found BGT enzymes (GPI-anchored glycoside hydrolases) with a role in cell wall remodeling at fusion pores (Fig. 4B) and at septal pores (Fig. 2) with a similar distribution to CSR-3 and CHS-2.

Thank you for pointing this out — we had indeed overlooked it. We have now included this finding in the discussion section and cited the corresponding reference

- Line 657: Correct "Glukanex" to "Glucanex"

This has been fixed

- It is hard to picture how cell wall biosynthesis is required for the formation of a stable fusion pore. A scheme illustrating the different steps leading to the pore formation and stabilization would be very helpful.

While we find this idea very interesting, speculating on the individual steps is premature given the current data. We believe that cell wall degradation and synthesis occur simultaneously to maintain balanced cell wall remodeling. However, since the exact architecture and sequence of molecular events remain unknown, presenting a model as a schematic could be misleading. Therefore, we have decided not to include such an illustration.

Reviewer #3:

Discussion-
Line 467: I guess it is a question whether one can say deletion in CSR-3 results in frequent cell fusion defects. Clearly, with reduced Ca2+ the defect becomes more apparent, but since the lysis rates are relatively moderate, I still question how severe the consequences are.

Thank you for pointing this out! What we actually wanted to say was "more frequent" instead of "frequent". This issue has been fixed.

Methods-
Some more details would be useful for the Southern Blotting validation. For example, what sequence was used as a probe? I assume some sort of PCR product of csr-3?

This information has been added to the figure legend
Also, some small typos I noticed:

Figures:
Figure S3C, extra x-axis labels present?

The issue was that the long genotype description spanned two lines, which could give the impression of additional labels. We adjusted the spacing between the individual labels to clearly indicate which label corresponds to which element.

line 150: CDSAERCH

has been fixed

line 161: created using a gene replacement strategy

has been fixed

line 250: conidiophors

has been fixed

line 335: missing word, should this say "a CSR-3 version"?, also it probably helps others if you specify the amino acid position of the cysteine

has been fixed and information has been added

line 350: phosphoproteome, also in caption for Figure S2A

has been fixed

line 424: I would hesitate to say "perfect", since the overlap is not literally 100%

we agree and removed the term "perfect"

line 434: likewise, I would hesitate to say "identical", maybe instead "indistinguishable"

we agree and edited the text accordingly

line 464: This sentence is current tense, but when referring to Dudin et al (Line 457) that sentence is in past tense.

We believe our wording is correct. We state that “Dudin et al. demonstrated that during fission yeast mating, hydrolases are very precisely delivered to the fusion point via actin and myosin V,” referring to a past experimental finding. We then note that “the authors hypothesize that in the surrounding area…,” which is still an active hypothesis. Therefore, using the present tense for the hypothesis is appropriate.

line 657: glucanex

this has been fixed

Journal Requirements

1) We ask that a manuscript source file is provided at Revision. Please upload your manuscript file as a .doc, .docx, .rtf or .tex. If you are providing a .tex file, please upload it under the item type u2018LaTeX Source Fileu2019 and leave your .pdf version as the item type u2018Manuscriptu2019.

we uploaded the manuscript accordingly

2) We noticed that you used the phrase 'data not shown' in the manuscript. We do not allow these references, as the PLOS data access policy requires that all data be either published with the manuscript or made available in a publicly accessible database. Please amend the supplementary material to include the referenced data or remove the references.

we removed the reference

3) We do not publish any copyright or trademark symbols that usually accompany proprietary names, eg ©, ®, or TM (e.g. next to drug or reagent names). Therefore please remove all instances of trademark/copyright symbols throughout the text, including:
- ® on pages: 12, and 28
- TM on page: 28.

this has been fixed

4) Please upload all main figures as separate Figure files in .tif or .eps format. For more information about how to convert and format your figure files please see our guidelines: https://journals.plos.org/plosgenetics/s/figures

we uploaded all figures accordingly

5) We have noticed that you have uploaded Supporting Information files, but you have not included a list of legends. Please add a full list of legends for your Supporting Information files after the references list.

we added this information to the manuscript

6) Some material included in your submission may be copyrighted. According to PLOSu2019s copyright policy, authors who use figures or other material (e.g., graphics, clipart, maps) from another author or copyright holder must demonstrate or obtain permission to publish this material under the Creative Commons Attribution 4.0 International (CC BY 4.0) License used by PLOS journals. Please closely review the details of PLOSu2019s copyright requirements here: PLOS Licenses and Copyright. If you need to request permissions from a copyright holder, you may use PLOS's Copyright Content Permission form.
Please respond directly to this email and provide any known details concerning your material's license terms and permissions required for reuse, even if you have not yet obtained copyright permissions or are unsure of your material's copyright compatibility. Once you have responded and addressed all other outstanding technical requirements, you may resubmit your manuscript within Editorial Manager.

None of our material is copyrighted

Potential Copyright Issues:

i) Please confirm (a) that you are the photographer of 10B, S3, S6, and S11, or (b) provide written permission from the photographer to publish the photo(s) under our CC BY 4.0 license.

We confirm that all photos were taken by the authors of this manuscript, only for this manuscript and have not been published elsewhere before

ii) Figures 2C, 2E, and 3F. Please confirm whether you drew the images / clip-art within the figure panels by hand. If you did not draw the images, please provide (a) a link to the source of the images or icons and their license / terms of use; or (b) written permission from the copyright holder to publish the images or icons under our CC BY 4.0 license. Alternatively, you may replace the images with open source alternatives. See these open source resources you may use to replace images / clip-art:
- https://commons.wikimedia.org
- https://openclipart.org/.

We confirm that all drawings and illustrations of this manuscript were done by the authors of this manuscript, only for this manuscript and have not been published elsewhere before
